# Effect of amlodipine and lisinopril on microalbuminuria in patients with essential hypertension: A prospective study

**DOI:** 10.4103/0971-4065.62090

**Published:** 2010

**Authors:** S. Jalal, F. A. Sofi, S. M. Abass, M. S. Alai, M. A. Bhat, H. A. Rather, N. A. Lone, M. A. Siddiqi

**Affiliations:** Department of Cardiology, Sher-i-Kashmir Institute of Medical Sciences, (SKIMS), Soura, Srinagar, Kashmir, India; 1 Department of Internal Medicine, Sher-i-Kashmir Institute of Medical Sciences, (SKIMS), Soura, Srinagar, Kashmir, India; 2 Department of Nephrology, Sher-i-Kashmir Institute of Medical Sciences, (SKIMS), Soura, Srinagar, Kashmir, India; 3 Department of Immunology and Molecular Medicine, Sher-i-Kashmir Institute of Medical Sciences, (SKIMS), Soura, Srinagar, Kashmir, India

**Keywords:** Angiotensin converting enzyme inhibitors, calcium channel blockers, hypertension, microalbuminuria

## Abstract

Microalbuminuria can be present in 25–100% of patients with essential hypertension and is associated with increased incidence of cardiovascular events. Our goal was to evaluate the effect of a commonly used calcium channel blocker, amlodipine, and an angiotensin converting enzyme inhibitor, lisinopril on urinary albumin excretion in patients with mild to moderate essential hypertension. We screened 324 patients with essential hypertension for microalbuminuria and documented it in 120 patients. These 120 patients with microalbuminuria were randomly divided into two groups of 60 each, matched for age, sex, arterial pressure, creatinine clearance, and urinary albumin excretion so as to receive amlodipine or lisinopril. We prospectively measured their urinary albumin excretion and creatinine clearance prior to treatment and, four and eight weeks after treatment with amlodipine or lisinopril. Mean arterial pressure (mean ± SD) at baseline, after four weeks, and after eight weeks was 113.01 ± 4.38,104.93 ± 3.12, and 98.89 ± 1.75 mmHg (*P* < 0.0000); and 114.13 ± 7.11, 106.52 ± 3.50, and 100.89 ± 2.80 mmHg (*P* < 0.0000) in amlodipine and lisinopril groups, respectively. Urinary albumin excretion (mean ± SEM) at baseline, after four, and after eight weeks was 79.30 ± 3.74, 62.03 ± 3.61, and 52.02 ± 3.05 (*P* < 0.0000); and 73.96 ± 4.10, 72.39 ± 3.74, 66.12 ± 3.94 (*P* = 0.1742) in lisinopril and amlodipine groups, respectively. Lisinopril but not amlodipine, reduced the urinary albumin excretion significantly despite their similar antihypertensive efficacy. The clinical and prognostic significance of these observations need to be established.

## Introduction

The conventional methods of detecting renal damage in hypertension which include the measurement of blood urea nitrogen and creatinine, and proteinuria, are relatively insensitive, and only show abnormalities when the disease process is fairly advanced. Essential hypertension (EHT) produces clinical proteinuria and significant reduction in renal function in 5-15% of patients.[1] There has recently been considerable interest in the quantitative measurement of albuminuria to detect the subtle effects of hypertension on the kidneys. The term microalbuminuria was first used to describe the subclinical elevation of urinary albumin in patients with diabetic nephropathy. The advent of more sensitive method to quantitate the urinary albumin excretion (UAE) has unfolded higher frequency (25-100%) of microalbuminuria in patients with hypertension than in normotensive population.[2–6]

Several epidemiological studies have shown that proteinuria as well as microalbuminuria are independent predictors of cardiovascular morbidity and mortality in patients with EHT.[7] The hypothesis that reduction of UAE during long term antihypertensive treatment may induce an improvement in the cardiovascular complications associated with EHT, is still under investigation. The angiotensin converting enzyme (ACE) inhibitors have been known to induce a regression of the proteinuria both in normotensive and hypertensive patients with diabetic renal illness[8] and in hypertensive patients with chronic renal failure.[9] Calcium Channel Blockers (CCB) have only been evaluated recently with regard to their anti-proteinuric effects in humans with diabetes mellitus. Numerous short term studies have been performed in the past few years to assess the effects of different classes of CCB in the diabetes[10–14] and hypertension.[15,16] However, these studies revealed divergent results.

Since 25% of patients with end-stage renal disease (ESRD) have hypertension as the primary diagnosis[1] and moreover, microalbuminuria reflects endothelial damage which has been associated with increased cardiovascular mortality and morbidity, it becomes of paramount importance to study the effects of commonly used antihypertensive drugs like ACE inhibitor lisinopril and CCB-amlodipine on microalbuminuria and renal function in hypertension.

## Materials and Methods

We screened 324 patients with EHT, who attended General Medicine and Cardiology out-patient clinics of tertiary care hospital in north India. Hypertensive patients were included in the study if they met the following criterion.

- Diastolic blood pressure (DBP) persistently between 90 and 100 mmHg on three successive visits to the outpatient clinic.
- UAE was between 30-300 mg/24 hrs; we chose the level of 30 mg/24 hrs to define the presence of microalbuminuria because in a previous study it was shown that all the normal subjects had UAE below this value.[2]
- A creatinine clearance (Clcr) level greater than 80 ml/ min/l.73 m^2^ in three different measurements.
- No antihypertensive therapy in the past.

We enrolled 120 patients who met the above criteria. Females on birth control pills were excluded from the study. The diagnosis of secondary hypertension was excluded with regular laboratory analysis. Patients with clinical or laboratory evidence of hepatic, renal, thyroid or any other major illness like diabetes mellitus were excluded. This group of 120 patients was randomly divided into two subgroups of 60 each matched for age, sex, arterial pressure, Clcr and UAE. One subgroup received an ACE inhibitor lisinopril, other received a CCB amlodipine. The dose of drugs was gradually increased to maximum desirable dose.Aimed target blood pressure was <130/80 mmHg. The administration of the drugs was open labelled, and these drugs were administered at 8 am daily except on the day of study when the drugs were given only after blood pressure measurement and drawing of sample. 24-hour urine was collected before the start of the study and after four and eight weeks of treatment to measure UAE, creatinine, sodium and potassium. At baseline, and after four and eight weeks of treatment during the study creatinine clearance, serum sodium and potassium were also measured. Blood and urine creatinine were estimated by autoanalyser. Urinary albumin excretion was estimated by an immunoturbidometry method using an automated Biochemistry Analyzer (Hitachi704).

Statistical analysis was performed by using statistical package for social science (SPSS) version 11.5 for PC windows. A 2-tailed *P* value of < 0.05 was taken as statistically significant.

## Results

Patients on amlodipine or lisinopril did not differ with respect to their age, sex, BMI, smoking, and severity of HT [Table 1]. Also their baseline laboratory parameters were comparable [Table 2]. The mean daily dose of amlodipin used was 8.04 ± 2.06 mg (range, 5–10 mg). None of the patients on amlodipine experienced any adverse drug effect. In lisinopril group, mean daily dose of drug used was 8.21 ± 1.99 mg (range, 5-10 mg). One patient experienced mild dry cough which did not necessitate to stop the drug. The aimed target blood pressure (<13/80) was achieved in 81 (67.5%) patients (which included 42 (70%) and 39 (65%) in amlodepin and lisinopril group respectively). Baseline systolic (SBP), diastolic (DBP) and mean arterial pressures (MAP) were comparable between amlodipine and lisinopril group [Table 3]. There was significant reduction in SBP, DBP and MAP at 4 and 8 weeks in both the groups (*P* < 0.0000). We observed significant reduction in DBP and MAP after four and eight weeks of therapy in amlodipine group compared to lisinopril group (*P* = 0.0003, 0.025; and 0.01, <0.0001, respectively). However, SBP was comparable between the two groups at four and eight weeks of therapy [Table 3].

**Table 1:** Comparison between clinical characteristics of hypertensive patients on amlodipine and lisinopril

Parameter	Amlodipine group N = 60	Lisinopril group N = 60	*P* value
Age (years)	55.12 ± 4.85	53.23 ± 6.24	> 0.05
Range	(48-61)	(44-63)	
Sex			
Male (%)	27 (45)	33 (55.55)	> 0.1
Female (%)	33 (55)	27 (45)	
BMI (kg/m2)	24.43 ± 3.70	25.69 ± 4.13	> 0.05
Range	(20.98-31.56)	(18.46-32.87)	
Smoker (%)	33 (55)	27 (45)	> 0.01
Male (%)	20 (60.60)	14 (51.85)	
Female (%)	13 (39.40)	13 (48.15)	
Severity of hypertension*			> 0.1
Mild (%)	16 (26.67)	21 (35)	
Moderate (%)	44 (73.33)	39 (65)	

BMI = Body mass index

* Mild-140-149/90-94; Moderate-150-159/95-100mmHg; Values expressed as mean ± SD

**Table 2:** Laboratory characteristics of patients on amlodipine and lisinopril

Parameter	Amlodipine group n = 60	Lisinopril group n = 60	*P* value
Hemoglobin (gm/dl)	12.14 ± 2.02	12.25 ± 2.01	0.7
	(9.8-15.5)	(10.4-14.5)	
Bloodglucose (mg/dl)	88.11 ± 17.95	90.33 ± 22.93	0.5
	(68-110)	(66-124)	
Cholesterol (mg/dl)	160.77 ± 29.89	165.22 ± 33.26	0.4
	(106-200)	(103-200)	
Uric acid (mg/dl)	4.77 ± 0.94	4.58 ± 0.87	0.2
	(3.8-6.4)	(3.0-6.1)	
Proteins (gm/dl)	7.39 ± 0.94	7.19 ± 0.78	0.2
	(5.8-8.5)	(5.8-8.4)	
Albumin (gm/dl)	4.11 ± 0.64	4.09 ± 0.55	0.8
	(3.0-5.1)	(3.1-5.0)	

Values expressed as mean ± SD, unless specified; Figures in parentheses indicate ranges

**Table 3:** Baseline systolic, diastolic and mean arterial blood pressure prior to and after four and eight weeks of treatment with amlodipine and lisinopril in hypertensive microalbuminuric patients

	Baseline	4 weeks	8 weeks	*P* value
SBP (mmHg)				
Amlodipine group (n = 60)	149.66 ± 10.93	139.22 ± 9.60	131.66 ± 6.40	<0.0001
	(140-160)	(130-150)	(125-142)	
Lisinopril group (n = 60)	151 ± 11.86	138 ± 7.52	132.22 ± 7.76	<0.0001
	(142-160)	(132-150)	(125-140)	
	*P* < 0.524	*P* = 0.444	*P* = 0.669	
DBP (mmHg)				
Amlodipine group (n = 60)	94.69 ± 4.27	87.89 ± 3.64	84 ± 2.45	<0.0001
	(90-100)	(80-92)	(82-90)	
Lisinopril group (n = 60)	95.63 ± 4.87	90 ± 2.46	85.22 ± 3.23	<0.0001
	(90-100)	(80-92)	(84-92)	
	*P* = 0.267	*P* = 0.0003	*P* = 0.025	
MAP (mmHg)				
Amlodipine group (n = 60)	113.01 ± 4.38	104.93 ± 3.12	98.89 ± 1.75	<0.0001
	(106.67-120)	(96.67-111.30)	96.33-107.33)	
Lisinopril group (n = 60)	114.13 ± 7.11	106.52 ± 3.50	100.89 ± 2.80	<0.0001
	(107.33-120)	(97.33-113.33)	(97.66-108)	
	*P* = 0.305	*P* = 0.01	*P* = 0.0000	

Data expressed as mean ± SD; Figures in parentheses indicate range

Serum creatinine, serum sodium, serum potassium, and urinary sodium, and potassium levels were comparable between amlodipine group and lisinopril group prior to treatement, four and eight weeks after the treatment. These parameters did not change significantly over eight weeks of therapy with amlodipine or lisinopril [Table 4], we did not observe any significant change in Clcr after four and eight weeks of therapy with amlodipine or lisinopril compared to the baseline values [Table 5]. Also comparison of Clcr between two groups did not reveal any significant difference after four and eight weeks of treatment.

**Table 4:** Effect of amlodipine and lisinopril on various laboratory parameters in microalbumiuric patients with essential hypertension

	Baseline	4 weeks	8 weeks	*P* value
Serum creatinine (mg/dl)				
Amlodipine group (n = 60)	0.88 ± 0.13	0.87 ± 0.20	0.86 ± 0.15	0.4
	(0.58-1.02)	(0.56-1.07)	(0.58-1.03)	
Lisinopril group (n = 60)	0.96 ± 0.22	0.91 ± 0.15	0.90 ± 0.14	0.130
	(0.74-1.42)	(0.72-1.06)	(0.66-1.02)	
Serum soeidum (mmol/L)				
Amlodipine group (n = 60)	141.23 ± 6.45	141.89 ± 5.70	139.57 ± 5.29	0.13
	(128-152)	(129-1500	(143-148)	
Lisinopril group (n = 60)	139 ± 5.42	140.57 ± 5.09	138.45 ± 3.52	0.51
	(129-144)	(132-150)	(129-140)	
Serum potassium (mmol/L)				
Amlodipine group (n = 60)	3.93 ± 0.49	3.77 ± 0.52	3.88 ± 0.58	0.6
	(3.2-5.0)	(3.0-4.2)	(3.2-4.3)	
Lisinopril group (n = 60)	4.11 ± 0.59	3.90 ± 0.58	4.12 ± 0.67	0.9
	(3.2-4.81)	(3.0-4.8)	(3.2-4.3)	
Urinary sodium (mmol/L)				
Amlodipine group (n = 60)	143.94 ± 11.73	143.46 ± 10.39	146.52 ± 11.90	0.23
	(121-156)	(118-145)	(132-162)	
Lisinopril group (n = 60)	142.85 ± 11.70	144.52 ± 6.35	144.30 ± 8.40	0.4
	(125-160)	(135-153)	(129-155)	
Urinary potassium (mmol/L)				
Amlodipine group (n = 60)	46.80 ± 14.14	47.24 ± 5.74	50.24 ± 7.35	0.10
	(29-56)	(40-56)	(39-60)	
Lisinopril group (n = 60)	49.24 ± 8.84	47.91 ± 8.61	52.69 ± 9.62	0.06
	(38-64)	(38-60)	(30-90)	

Data expressed as mean ± SD; Figures in parentheses indicate range; Amlodepin *vs.*lisinopril group: *P* value for all parameters at base line, four and eight weeks > 0.08 (not significant)

**Table 5:** Effect of 4 and 8 weeks treatment with amlodipine and lisonopril on creatinine clearlance and urinary albumin excretion in essential hypertension patients with microalbuminuria

	Baseline	4 weeks	8 weeks	*P* value
ClCr (ml/min)				
Amlodipine group (n60)	93.98 ± 6.40	93.43 ± 5.97	92.46 ± 6.81	0.214
	(85-103)	(85-102)	(83-103)	
Lisinopril group (n60)	93.21 ± 7.49	92.96 ± 7.30	93.90 ± 8.06	0.6307
	(83-105.1)	(84-102)	(83.1-106.81)	
	*P* 0.5497	*P* 0.7027	*P* 0.2968	
UAE (mg/24 hr)				
Lisinopril group (n60)	79.3 ± 3.74*	62.03 ± 3.61*	52.02 ± 3.05*	<0.0001
	(35.9-120.2)	(35.9-120.2)	(32.2-110.3)	
Amlodipine group (n60)	73.96 ± 4.10*	72.39 ± 3.74*	66.12 ± 3.94*	0.1742
	(38.7-131.1)	(38.7-131)	(38.1-132)	
	*P* 0.3420	*P* <0.047	*P* <0.0059	

* Standard error of mean; Data expressed as mean ± SD; Figures in parentheses indicate range

There was a significant reduction in UAE rate after four and eight weeks of treatment with lisinopril, compared to baseline value (*P* = 0.0000). In amlodipine group, there was no significant reduction in UAE rate after four and eight weeks of therapy compared to baseline value (*P* > 0.1742) [Table 5].

We observed significant reduction of UAE rate in lisinopril group after four weeks (*P* < 0.04) and eight weeks (*P* < 0.005) of therapy compared to that of amlodipine group [Table 5].

## Discussion

The passage of various substances including albumin, through the glomerulus, not only depends upon their molecular size, electrical charge, and shape but also to the size and selective properties of the glomerular filtrate. Renal hemodynamics also plays an important role.[17] The transport of albumin across the glomerular filter is influenced by the variations in the glomerular hemodynamics seen in essential hypertension. In fact, the renal alterations that accompany arterial hypertension are characterized by an increase in renal vascular resistance, a decrease in renal flow, and the initial retention of glumerular filtrate which will tend to diminish only at a later stage. The slight alteration in the glomerular filtrate is probably caused by vasoconstriction of the efferent arteriole, which in turn is responsible for intraglomerular hypertension and for the increased filtration fraction.[18] This process is definitely regulated by neurohormonal mechanisms such as the vascular response to the noradrenaline and angiotensin.[19] Therefore, it is highly probable that through vasoconstriction efferent arteriole,angiotensin II increases the hydrostatic pressure in the glomerular capillaries to retain the glomerular filtrate, which results, however, in an even greater filtration of proteins. Therefore, the proteinuria could be a result not only of structural modifications of the glomeruli but also of these hemodynamic alterations.

Theoretically, proteinuria, in the subject with hypertension, can be diminished by way of regression of the structural renal alterations or modifications of the hemodynamics of the afferent arteriole, glomerulus, efferent arteriole system or through mechanisms that re-establish normal glomerular permeability.[20] The results of our study clearly demonstrate that despite similar antihypertensive efficacy, lisinopril but not the amlodipine, reduced UAE significantly in patients with EHT and microalbuminuria. These observations are consistent with previous studies.[21,22] Amlodipine has also been compared to eplerinone—a selective aldosterone blocker—in patients with systolic hypertension and found inferior in reducing microalbuminuria (10% *vs.* 52%) despite good and comparable blood pressure control.[23]

In previous studies conducted both on animals and human, ACE inhibitors were shown to be able to reduce urinary excretion of proteins in various types of renal disease including those associated with diabetes.[8,9,24] The mechanisms by which ACE inhibitors confer a beneficial effect on proteinuria reduction are largely unknown. The systemic arterial pressure can play an important role by reducing the filtration pressure. However, no correlation has been observed between antihypertensive activity and the effects on proteinuria.[16] Furthermore, the reduction in albumin excretion that we observed after lisinopril does not seem to be necessarily connected to the antihypertensive properties of the drug because amlodipine, which has the same antihypertensive activity and even better control of DBP and MAP than lisinopril, had no significant effect on microalbuminuria. Various mechanisms have been proposed to explain the beneficial effects of ACE inhibitors on proteinuria. The first is that this phenomenon might be due to the improvement in intrarenal hemodynamics. The second mechanism is that this category of drugs can reduce the permeability of the basement membrane of the glomerulus. The first hypothesis derives primarily from studies that show how ACE inhibitors diminish proteinuria, the renal filtration fraction, and the intraglomerular pressure in various experimental models of renal disease.[24,25] Since angiotensin II increases the tone of the efferent arteriole, the intraglomerular pressure, and the proteinuria, it is conceivable that ACE inhibitors can reduce the proteinuria by inhibiting the effect of angiotensin II in renal microcirculation. Furthermore, some experts have recently affirmed that ACE inhibitors can diminish proteinuria by reducing the permeability of the basement membrane of the glomerulus.[26]

Microalbuminuria in EHT reflects systemic dysfunction of vascular endothelium, a structure intimately involved in the permeability, hemostasis, fibrinolysis, and blood pressure control. Inhibition of angiotensin II conversion and preservation of nitric oxide production are considered to underlie the favorable effects of ACE inhibition on endothelial function and potentially on cardiovascular events. Both angiotensinII and nitric oxide are involved in the balance of thrombosis, and fibrinolysis, via changes in platelet aggregation plasminogen activator, as well as changes in the matrix synthesis of plaques.[27,28] Hence, it is conceivable that reduction in microalbuminuria by ACE inhibitor is likely due to its favorable effect on vascular endothelium.

We did not observe any significant modifications in microlbuminuria with amlodipine. Calcium acts as a second messenger to mediate the vasoconstriction effect of angiotensin and nor epinephrine. If, in subjects with hypertension, the efferent arteriole is further constricted by these hormonal regulators, then a positive response by calcium antagonists appears justified. The modifications of hemodynamics with calcium antagonists are related to different factors, such as, the type of calcium antagonist, the way in which it is administered, basal vascular tone, and the basal levels of vasoconstrictors, such as angiotensin II.[10] In this study, amlodipine administered orally did not alter UAE significantly. This might be attributable either to poor selectivity of the drug for renal vessels or to the fact that in these patients the drug acted more on the afferent arteriole than on efferent arteriole leaving the intraglomerular hypertension unchanged furthermore, it seems unlikely that it can act on the permeability of the basement membrane, given the total absence of significant changes in the UAE.

We did not observe any change in creatinine clearance in hypertensive patients treated with lisinopril or amlodipine. Similar results have been observed in the past.[21,29]

Since microalbuminuria in EHT reflects systemic vascular endothelial dysfunction, its reduction by ACE inhibitor lisinopril points towards the favorable effects of ACE inhibition on vascular endothelium. Recently, HOPE trial has also revealed beneficial effects of ACE inhibitors on vascular endothelium resulting in reduction of cardiovascular morbidity and mortality.[30] The implications of this observation on cardiovascular mortality and morbidity, and in future, progression of renal disease in patients with EHT remain to be established.

In conclusion, this study has shown that eight weeks of therapy with lisinopril reduces UAE in patients with mild to moderate EHT, whereas amlodipine had no measurable effect on UAE, despite similar antihypertensive efficacy.
